# Electrochemical interactions in the oral cavity: The clinical significance of oral galvanism – a narrative review

**DOI:** 10.1097/MD.0000000000051445

**Published:** 2026-11-06

**Authors:** Tushar Khanna, Nakul Sharma, Ashish Jain, Gurparkash Singh Chahal, Vishakha Grover, Adel Saeed N. Alobaid, Anshad M. Abdulla, Suraj Arora, Gotam Das, Priyanka Saluja, Waled Abdulmalek Alanesi

**Affiliations:** a Dr. Harvansh Singh Judge Institute of Dental Sciences and Hospital, Panjab University, Chandigarh, India; b Department of Periodontology and Oral Implantology, Dr. Harvansh Singh Judge Institute of Dental Sciences & Hospital, Panjab University, Chandigarh, India; c Department of Restorative Dental Sciences, College of Dentistry, King Khalid University, Abha, Saudi Arabia; d Department of Paediatric Dentistry & Orthodontic Sciences, College of Dentistry, King Khalid University, Abha, Saudi Arabia; e Department of Prosthodontics, College of Dentistry, King Khalid University, Abha, Saudi Arabia; f Mike Petryk School of Dentsitry, University of Alberta, Edmonton, Alberta, Canada; g Department of Operative Dentistry, Faculty of Dentistry, University of Science and Technology, Inmaa City, Aden, Yemen.

**Keywords:** galvanic corrosion, implant corrosion, metal ion release, multimetal restorations, oral galvanism, pathogalvanism

## Abstract

Electrochemical reactions in the oral cavity, caused by small-scale electric charges between different metallic prosthetic materials, are often overlooked in modern dentistry. Although multiple metallic alloys are frequently used in dentistry, their unintentional interactions may lead to both localized tissue effects and have been hypothesized to contribute to broader biological consequences. However, awareness of these interactions remains insufficient among dental practitioners and patients. A narrative review of the literature was conducted using electronic databases including PubMed/MEDLINE, Scopus, Web of Science, Google Scholar, and Embase. Relevant studies addressing the etiology, pathophysiology, clinical manifestations, diagnosis, prevention, and management of oral galvanism were identified and qualitatively synthesized. The reviewed literature indicates that electrochemical interactions between metallic dental materials can result in galvanic current generation, corrosion processes, metal ion release, and a variety of oral manifestations. Previous reports have suggested that more than 30 different metallic components may coexist within a single oral cavity, highlighting the complexity of oral electrochemical interactions. While several studies have proposed potential associations with broader biological and systemic effects, the available evidence remains limited and heterogeneous. Recognizing the effects of electrochemical activity in the oral cavity is essential for enhancing biocompatibility, ensuring patient safety, and improving the longevity of dental restorations. This review seeks to address existing knowledge gaps and promote further research into this often-overlooked aspect of dental health.

## 1. Introduction

First identified by Sulzer et al in 1754, oral galvanism refers to the electrochemical corrosion of metallic dental restorations caused by the continuous flow of electric charge in a saliva-rich environment where 2 or more different metals coexist.^[1]^

A galvanic current, also called a bimetallic cell, forms in the oral cavity when metals with different electrochemical properties interact in the presence of an electrolyte, such as saliva. Dental materials composed of metals such as titanium, cobalt, nickel, gold, palladium, mercury, iron, silver, and copper exhibit unique electrochemical characteristics, leading to variations in their electrical potential.^[1–3]^

The interaction of these metals within the oral cavity can create unintended electromotive currents, a phenomenon often compared with a miniature battery effect within dental restorations.^[4]^ Electrochemical corrosion occurring within the oral cavity results in the gradual release of metal ions from restorations, which may contribute to localized tissue irritation, while potential systemic effects remain an area of ongoing investigation. When 2 dissimilar metals are exposed to an electrolyte such as saliva or tissue fluids, a localized electrochemical reaction occurs, promoting metal ion release from dental alloys.^[5,6]^ Even at low concentrations, released metal ions may contribute to both immediate and long-term changes in the oral environment, including corrosion-related degradation and tissue responses.^[1,7]^

Symptoms associated with oral galvanism, also known as pathogalvanism, range from a persistent metallic taste and tingling sensations to burning discomfort and inflammatory reactions, which are frequently reported among affected individuals.^[6,8]^

Although the precise prevalence of oral galvanism remains difficult to determine because of variations in diagnostic criteria, underreporting, and the nonspecific nature of symptoms, its clinical significance has increased with the widespread use of metallic restorations and fixed prostheses, including dental implants. Recognition of oral galvanism is important because it may contribute to patient discomfort, corrosion-related deterioration of restorative materials, and diagnostic challenges arising from symptom overlap with other oral conditions.

There are multiple scenarios in which oral galvanism can occur, each influenced by the placement and interaction of different metallic restorations in the oral cavity.

A classic example occurs when a silver amalgam filling is placed adjacent to or in contact with a gold restoration. The combination of dissimilar metals in the presence of saliva and tissue fluids forms a small electrical circuit, which may generate a galvanic current. This current can travel through the dental pulp, potentially leading to mild discomfort or pain.^[9,10]^ Galvanic currents can also be induced between 2 teeth in the same arch, even when they do not directly touch. The electrical potential difference between metallic restorations on separate teeth may still result in a low-level electric charge, affecting the surrounding tissues.^[11]^ When a patient bites down, opposing restorations made of different metals can momentarily come into direct contact, leading to electrical discharge. This can result in sharp pain or discomfort, particularly in patients with highly sensitive pulp tissue.^[12,13]^ Two neighboring teeth restored with dissimilar metals may also create a galvanic couple. In this case, electricity flows from one metal to another through the dental tissues, including the dentin, bone, and tissue fluids, potentially triggering localized irritation or pain.^[14]^

The aim of this narrative review is to provide a comprehensive overview of oral galvanism by summarizing its etiology, pathophysiological mechanisms, clinical manifestations, diagnostic approaches, preventive strategies, and management options, while highlighting current concepts and emerging areas of research.

## 2. Methodology

This narrative review was conducted to summarize the current evidence regarding oral galvanism, including its etiology, pathophysiological mechanisms, clinical manifestations, diagnostic approaches, preventive strategies, and management options. A comprehensive literature search was performed in electronic databases including PubMed/MEDLINE, Scopus, Web of Science, Google Scholar, and Embase.

The search strategy used combinations of Medical Subject Headings terms and free-text keywords that include “oral galvanism,” “oral galvanic phenomena,” “galvanic corrosion,” “electrochemical interactions,” “oral electrochemistry,” “dental alloys,” “metallic restorations,” “dissimilar metals,” “oral metallic taste,” “oral burning sensation,” “burning mouth syndrome,” “oral discomfort,” “titanium implants,” “dental implants,” “peri-implant disease,” “peri-implant oral malignancy,” “oral squamous cell carcinoma,” “oral carcinogenesis,” “biocorrosion,” “metal ion release,” “electrical currents,” “electrogalvanism,” “electromagnetic effects,” “oxidative stress,” “inflammation,” “immune response,” “hypersensitivity reactions,” “neurotoxicity,” “neurological manifestations,” “cognitive impairment,” “memory disturbances,” “chronic fatigue,” “sleep disturbances,” and “oral medicine.” Boolean operators (AND, OR) were used to combine search terms and optimize retrieval of relevant literature.

The study period extended from October 1979 to January 2025, and articles published in English during this period were considered for inclusion in this narrative review. Original research articles, clinical studies, observational studies, case reports, narrative reviews, systematic reviews, and relevant experimental investigations addressing oral galvanism or related electrochemical phenomena in the oral cavity were evaluated. Reference lists of selected articles were also screened to identify relevant publications.

Studies were selected based on their relevance to the objectives of the review. Publications focusing on electrochemical interactions between dental materials, the development of galvanic current with the process of corrosion and its biological implications, diagnostic methods, and management strategies were included. Articles unrelated to oral galvanism, studies lacking sufficient scientific relevance, conference abstracts without accessible full texts, and non-English publications were excluded.

The retrieved literature was critically examined by all authors and qualitatively analyzed. Findings were qualitatively interpreted in the context of the strength, consistency, and clinical relevance of the available evidence. Given the heterogeneous nature of the available evidence and the narrative design of the present review, a quantitative meta-analysis was not considered appropriate.

Ethical approval and informed consent were not required for this study because it is a narrative review based exclusively on previously published literature and did not involve human participants, patient data, or animal subjects.

## 3. Etiology

The development of oral galvanism is influenced by multiple factors, both dental and systemic in origin. This condition occurs due to electrochemical interactions between different metallic elements in the oral cavity, which can be intensified by various physiological and environmental conditions.^[15]^

### 3.1. Dental origin

Various oral factors contribute to galvanic activity, including the type and combination of restorative materials, surface characteristics of metals, dental plaque accumulation, and microbial influences. Certain microbial factors, including bacterial biofilms, may accelerate metal corrosion and increase the effects of galvanic activity in the oral cavity.^[1,16,17]^

Research has shown that bacterial species like *Streptococcus sanguis* contribute to corrosion by creating localized anodic and cathodic zones on metal restorations, promoting galvanic currents. This reaction releases various metabolic byproducts, such as organic acids, which directly interact with metal surfaces, accelerating corrosion.^[18]^ Biofilm formation plays a crucial role in metal corrosion, as bacterial colonies create microenvironments with varying oxygen levels and metal ion concentrations, accelerating electrochemical degradation.^[12,19]^

Oxygen concentration cells: a heterogeneous biofilm creates areas with varying oxygen levels, causing some metal surfaces to act as anodes and others as cathodes. *Streptococcus mutans*, an oxygen-consuming bacterium, influences oxygen distribution, leading to localized galvanic reactions.^[20,21]^ Metal ion concentration cells: bacteria produce extracellular polysaccharides, forming a polymer matrix that binds metal ions differently across the surface. Areas under colonies with low metal ion affinity behave as anodes, while those under high-affinity colonies behave as cathodes.^[12,22,23]^

Active-passive cells: some bacteria, such as sulfate-reducing bacteria like *Desulfovibrio* species, produce hydrogen sulfide (H_2_S), forming iron sulfide (FeS) deposits. This acts as a strong local cathode, accelerating metal degradation and biomaterial destruction.^[24,25]^ Microbial symbiosis also plays a role in corrosion. Aerobic sulfide-oxidizing bacteria oxidize sulfides to elemental sulfur, creating anaerobic zones that support sulfate-reducing bacteria. In addition, anaerobic pathogens such as *Fusobacterium nucleatum* and *Prevotella melaninogenica* produce butyric acid, carbon dioxide, and hydrogen, further contributing to metal corrosion.^[12,26,27]^

While local electrochemical and microbiological factors play a central role in the initiation and progression of oral galvanism, the clinical expression of the condition is also influenced by patient-related systemic and environmental factors.

### 3.2. Nondental origins

Several systemic conditions and external factors influence the severity and perception of oral galvanism.

Systemic health conditions that alter salivary flow, pH balance, or mucosal sensitivity may increase a patient’s susceptibility to galvanic activity in the oral cavity. Postmenopausal women often exhibit reduced salivary production, shifts in pH, and increased mucosal sensitivity, making them more prone to discomfort caused by galvanic currents.^[28,29]^ Patients with gastroesophageal reflux disease may experience increased oral acidity, which can enhance the corrosion of metal restorations and amplify galvanic effects. In individuals with diabetes, neuropathic changes can heighten oral sensitivity, while fluctuations in salivary pH may contribute to increased metal corrosion and galvanic activity. Patients with hypothyroidism often experience altered taste, mouth burning, and decreased salivary pH, all of which can intensify oral galvanism symptoms.^[8]^ Changes in salivary conductivity, often seen in patients with dehydration, kidney disorders, or high physical exertion, can amplify the intensity of galvanic currents in the oral cavity. Autoimmune conditions can heighten metal hypersensitivity, worsening symptoms of oral galvanism. In addition, radiotherapy patients often experience increased salivary electroconductivity and poor mucosal healing, making them more vulnerable to metallic corrosion.^[8,30]^

Although often overlooked, psychosomatic factors play a crucial role in oral galvanism. Studies suggest that the condition resembles burning mouth syndrome (BMS) or atypical facial pain, with similar mental health disturbances observed in affected individuals. In one case, a patient’s symptoms nearly disappeared after receiving hormone therapy for postmenopausal disorders, indicating a potential hormonal link.^[7]^ Tobacco and alcohol use can compromise oral mucosal integrity, increasing the risk of metal corrosion and enhancing the effects of galvanic currents. Cigarette smoke may delay tissue healing, while alcohol can alter oral pH and accelerate metal degradation.^[8]^

## 4. Mechanism

The phenomenon of oral galvanism occurs when metallic dental restorations interact electrochemically within the oral cavity. This can happen either through direct contact between different metals or via the surrounding electrolyte, such as saliva, which contains charged ions like sodium, chloride, phosphate, and hydroxyl.^[30]^ Ionic components in saliva act as conductors, allowing the transfer of electrical charge between different metal surfaces, generating a galvanic current. Although some electrochemical interactions are benign, others can cause adverse effects, referred to as pathogalvanism, which may lead to localized discomfort and has been hypothesized to contribute to broader biological effects in susceptible individuals.^[6]^

The intensity of galvanic currents depends largely on the electrochemical potential differences between metals used in dental restorations. When 2 dissimilar metals are placed in the oral cavity, their varying reduction potentials create a galvanic cell, especially in the presence of saliva or other biological fluids acting as an electrolyte. Within this electrochemical reaction, the metal with a lower reduction potential functions as the anode, where oxidation occurs, while the metal with a higher reduction potential serves as the cathode, undergoing reduction (Fig. 1).^[3,13]^ As a result, metal ions are released into the surrounding tissues, which may provoke inflammatory responses, disrupt pH balance, and potentially influence microbial activity within the oral cavity. Moreover, the presence of electrical currents near implant surfaces may negatively affect osteointegration, particularly in titanium-based implants, by altering bone-implant interactions.^[13]^

**Figure 1.**
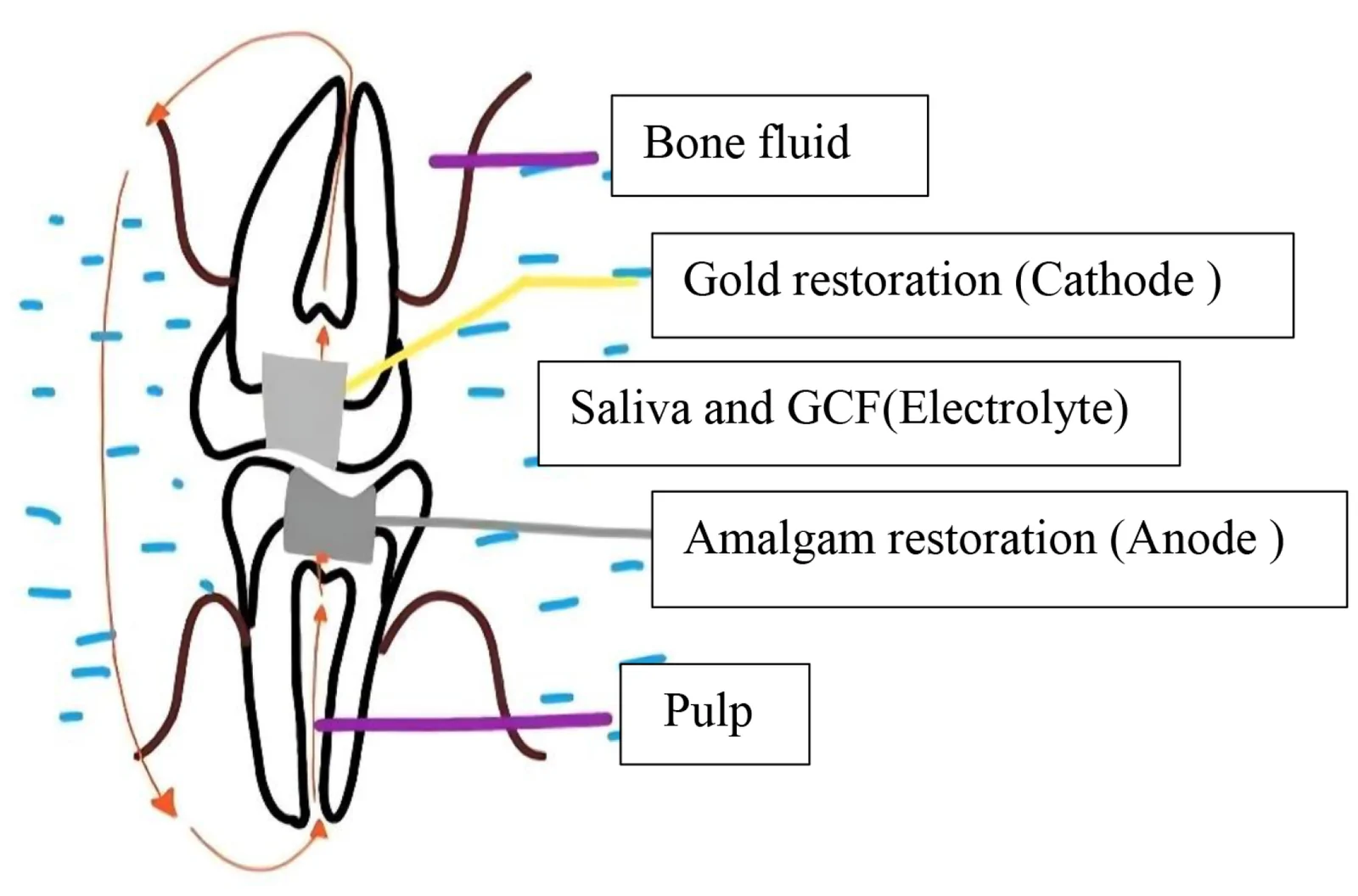
Schematic representation of galvanic current flow between dissimilar metallic restorations in the oral cavity. GCF = gingival crevicular fluid.

In clinical practice, galvanic currents may be detected in multiple intraoral situations, such as the interaction between dissimilar metal alloys, adjacent placement of amalgam restorations and metal crowns, or opposing metallic restorations generating electrical disturbances during mastication.^[31]^ Implant-supported prosthetic systems present a significant example, as components like titanium fixtures, abutments, and crowns interact at multiple junctions, increasing the likelihood of galvanic activity. Known as implant galvanic corrosion, this effect frequently occurs at the interface between the abutment and fixture, resulting in the gradual leaching of metal ions into the surrounding tissues.^[1]^

The diagram illustrates an amalgam restoration (anode) and a gold restoration (cathode) in the presence of saliva as the electrolyte. Electrons (e^−^) flow from the anode (amalgam) to the cathode (gold) through the metallic pathway, while ionic current flows through the saliva from the cathode to the anode. This electrochemical interaction results in corrosion of the less noble metal (amalgam), potentially leading to metal ion release, altered taste perception, and oral discomfort.^[32]^

The continuous movement of the tongue plays a role in galvanic activity by acting as a medium for electrical conduction. As it comes into contact with various metallic restorations, it facilitates the formation of temporary circuits in the presence of salivary electrolytes.^[33]^ As the tongue moves and interacts with different metallic surfaces, it can intermittently complete an electrical circuit, leading to the generation of brief galvanic currents, similar to a miniature electrochemical cell in the oral cavity.^[3]^ Galvanic activity can also arise due to variations in the composition and structure of different metal surfaces used in dental restorations. Certain dental materials, including eutectic and peritectic alloys, along with multiphase alloys such as amalgam, are more prone to galvanic corrosion due to their varied microstructural composition. Amalgam restorations contain multiple structural phases, with the tin-mercury (γ2) phase being particularly vulnerable to corrosion, especially in formulations with lower copper content.^[4]^ Over time, the accumulation of corrosion byproducts, such as oxides and chlorides of tin, copper, and silver, can degrade the structural integrity of the restoration.^[31]^

Oral galvanism functions similarly to an electrochemical cell, where the anode (e.g., a titanium implant), cathode (e.g., a metallic crown or filling), and electrolyte (saliva) contribute to the flow of electrical charges. The movement of electrical charge between these components promotes metal degradation, leading to the release of potentially harmful metal ions, which may cause tissue irritation and other biological responses depending on the concentration, duration of exposure, and host susceptibility.^[11]^ In addition, the presence of extracellular fluids such as blood and gingival crevicular fluid further complicates the electrochemical environment, increasing the likelihood of galvanic interactions.^[19]^ Reducing galvanic activity in the oral cavity requires strategic selection and proper placement of dental materials to minimize electrochemical interactions. Avoiding direct contact between dissimilar metals and minimizing prolonged exposure of metallic restorations to electrolyte-rich environments can help reduce the risk of galvanic corrosion and its associated clinical consequences.^[12]^

## 5. Factors determining oral galvanism

Several factors, including electrochemical properties of metals, material composition, and environmental conditions, contribute to the occurrence and intensity of oral galvanism. Galvanic corrosion in dental restorations is influenced by both intrinsic factors related to the materials themselves and extrinsic factors associated with the oral environment.

Electrochemical factors – galvanic corrosion is primarily governed by core electrochemical principles, including

Electrode potential: the voltage difference between 2 dissimilar metals determines the intensity of the galvanic current. The greater the difference, the higher the tendency for corrosion (Table 1).

**Table 1:** Electrochemical characteristics and galvanic interactions of dental materials.

Material	Electrode potential (V)	Corrosion resistance	Galvanic risk	Common clinical use	Best combination pairs
Gold (Au) Alloy	+1.42V	Excellent	Low	Crowns, inlays, bridges	Gold-Gold, Gold-Titanium
Titanium (Ti)	−1.63V	Very high	Low	Implants, prosthetic frameworks	Titanium-Titanium, Titanium-Gold
Cobalt-Chromium (CoCr)	−0.55V	High	Moderate	Metal frameworks, partial dentures, crowns	CoCr-CoCr, CoCr-Titanium
Nickel-Chromium (NiCr)	−0.80V	Moderate	High	Crowns, bridges	NiCr-NiCr (avoid noble metals)
Silver Amalgam	−0.71V	Moderate	High	Restorations	Amalgam-Amalgam (avoid Gold/Titanium)
Stainless Steel	−0.44V	Moderate	Moderate	Orthodontic wires, crowns	Stainless Steel-Stainless Steel
Zirconia (ZrO_2_)	N/A (ceramic)	Excellent	None	Crowns, bridges, implants	Zirconia-Zirconia, Zirconia-Titanium
Palladium (Pd) Alloy	+0.95V	High	Low	High-noble metal crowns, bridges	Palladium-Gold, Palladium-Palladium
Aluminum Oxide (Al_2_O_3_)	N/A (ceramic)	Excellent	None	Ceramic restorations	Al_2_O_3_- Al_2_O_3_, Al_2_O_3_-Zirconia

Table 1 outlines the corrosion resistance, galvanic risk, common clinical applications, and optimal material pairings to minimize electrochemical interactions in the oral environment.

The ability of an electrode to resist changes in potential influences the stability of the galvanic couple. The ratio of cathode-to-anode surface area impacts corrosion severity, with smaller anodes coupled to larger cathodes being particularly vulnerable. The overall resistance within the electrochemical circuit affects the magnitude of the current flow, influencing the rate of corrosion. Saliva composition, pH, and ion concentration significantly impact galvanic activity.

Variations in aeration and ion diffusion alter the corrosion rate.^[14]^

### 5.1. Intrinsic factors (material-dependent)

The composition of a dental alloy significantly affects its corrosion behavior. While commercially pure titanium (cp Ti) has high biocompatibility and corrosion resistance, it lacks mechanical strength. Titanium-Aluminum-Vanadium (Ti-6Al-4V) alloys offer improved mechanical properties but are more prone to galvanic corrosion. High-gold alloys exhibit superior corrosion resistance due to their thermodynamic stability, while palladium additions improve the durability of silver-based alloys. Alloys such as nickel-chromium (NiCr) and cobalt-chromium (CoCr) have higher dissolution rates, making them more susceptible to corrosion compared with noble metal alloys.^[9,13,16]^ The surface properties of dental materials, including oxide film thickness, roughness, and grain structure, affect their corrosion resistance. Titanium surfaces treated with acid etching or anodization exhibit improved resistance to galvanic corrosion and better osteointegration.^[21]^ Localized corrosion, such as pitting and crevice corrosion, can damage the passive oxide layer on metal surfaces, leading to increased vulnerability to galvanic interactions.^[11,17]^

### 5.2. Extrinsic factors (environment-dependent)

The electrochemical stability of dental materials is heavily influenced by pH fluctuations. A more acidic salivary environment enhances galvanic current flow, accelerating metal degradation and corrosion.^[9,18,24]^ While low fluoride concentrations (e.g., 227 ppm at pH 5.5) are generally safe for titanium, excessive fluoride levels in mouthwashes and gels can degrade its protective oxide layer, increasing the risk of corrosion.^[20]^ Proper material selection for implant-prosthetic structures is essential to minimize galvanic interactions. Using titanium-based prostheses on titanium implants significantly lowers the likelihood of electrochemical corrosion. Research suggests that optimal material pairings may include Cp Titanium + Titanium Alloy, Titanium + Gold, and Titanium + CoCr.^[28]^ The surface area ratio between anode and cathode plays a crucial role in corrosion rates. A smaller anode paired with a larger cathode leads to rapid anodic dissolution, increasing the risk of corrosion.^[31]^

## 6. Symptomatology

Metallic dental materials continuously interact with both the hard and soft tissues of the oral cavity. The oral environment, characterized by fluctuations in pH, temperature, and moisture, can induce electrochemical and mechanical alterations in metallic restorations, potentially resulting in discomfort and adverse tissue responses.^[5]^

Patients with oral galvanism commonly report a persistent metallic taste, burning sensations, alterations in taste or smell perception, and discomfort involving the tongue or oral mucosa.^[1,6]^ Symptoms may become more pronounced following occlusal adjustments or in the presence of adjacent or opposing dissimilar metallic restorations, such as amalgam fillings, metal crowns, or implant-supported prostheses.^[1]^

Prolonged galvanic activity has been associated with mucosal inflammation, asymptomatic metallic pigmentation around restorations, and varying degrees of oral discomfort ranging from mild irritation to persistent low-grade pain. Discomfort may arise either from electrochemical activity between metallic restorations or from transient contact between restorations and metallic objects during mastication.^[1,19,25]^

Studies suggest that galvanic currents exceeding 5 μA may contribute to intraoral inflammatory changes, particularly involving the buccal mucosa and tongue, and may be associated with glossodynia, neuralgia, BMS, and increased pulp hyperemia.^[1,5]^

## 7. Emerging and conflicting evidence

Several authors have proposed that prolonged exposure to galvanic phenomena within the oral cavity may contribute to the development of lichenoid lesions, particularly in tissues adjacent to metallic restorations or implant-supported prostheses. Long-term exposure to low-level electrical currents has also been suggested as a potential factor in the development of oral lesions, including oral lichen planus and verrucous leukoplakia. However, the available evidence remains limited and largely observational in nature.^[2,23,30]^

Higher galvanic current values have been associated with inflammatory and lichenoid changes, whereas lower values have been linked to asymptomatic metallic pigmentation around restorations.^[5]^ Oral lichen planus associated with metallic restorations may clinically present with characteristic Wickham’s striae involving the buccal mucosa and lateral borders of the tongue. In some cases, these lesions may exhibit erosive or erythematous changes and resemble other mucosal disorders, including leukoplakia and leukoedema.^[1]^

Leukoedema, characterized by the accumulation of swollen keratinocytes, has been proposed as a potential consequence of altered ionic concentrations generated by galvanic activity. It has been hypothesized that electrochemical changes within the oral environment may disrupt extracellular osmotic balance, resulting in cytoplasmic fluid loss, keratinocyte shrinkage, and superficial epithelial alterations. Similarly, erosive and ulcerative lesions associated with metal hypersensitivity may clinically mimic recurrent aphthous stomatitis.^[8]^

Several hypotheses have been proposed to explain the potential biological effects of oral galvanism. It has been suggested that metallic restorations may function as a weak battery in the oral cavity, producing ionic free radicals and accumulating ionic concentrations in the salivary buffer, especially adjacent to the buccal mucosa or the lateral surface of the tongue, and implant-related sulcus and junctional epithelium.^[11,14]^ These processes have been hypothesized to influence cellular behavior and tissue responses in adjacent mucosal tissues. Experimental observations have suggested possible effects on cellular proliferation, apoptosis, and inflammatory pathways; however, the clinical significance of these findings remains uncertain.

Potential links between oral galvanism and oral carcinogenesis remain controversial. Some authors have hypothesized that chronic electrochemical irritation, oxidative stress, and prolonged exposure to corrosion products may contribute to biological changes associated with oral potentially malignant disorders and oral squamous cell carcinoma. Nevertheless, current evidence is insufficient to establish a causal relationship, and the available data are derived primarily from experimental studies and limited observational investigations.^[1]^

Similarly, peri-implant oral malignancy (PIOM) has been proposed as a theoretical consequence of chronic electrochemical interactions occurring around implant-supported restorations. Experimental studies have reported cellular and subcellular alterations following exposure to direct electrical currents. For example, Wartenberg et al observed apoptosis in oral cancer cells exposed to direct-current electrical fields, whereas Podzimek et al reported altered proliferation and surface-marker expression in lymphocytes exposed to direct current. Chepelova et al further suggested that chronic galvanic currents may influence the biological behavior of oral lesions.^[2,5]^ While these findings provide mechanistic insights, their direct clinical relevance remains uncertain.

Voltage differences between dental alloys may reach several hundred millivolts, and galvanic currents within the oral cavity have been investigated in relation to tissue responses and mucosal irritation. Experimental models have demonstrated subcellular changes following electrical stimulation, leading some authors to hypothesize a potential role in PIOM and other biological effects. However, these observations should be interpreted cautiously, as they do not establish causation in clinical settings.^[1]^

Beyond local tissue effects, a limited number of studies have suggested possible associations between oral galvanism and broader neurological or systemic manifestations. Reports have described atypical neuralgic pain, headaches, facial discomfort, and, in rare cases, symptoms extending beyond the oral cavity, including lower back pain. Because many of these manifestations are nonspecific, they may occasionally be attributed to psychosomatic conditions or other underlying disorders.^[1]^ Fluctuating galvanic currents generated by intermittent contact between metallic restorations have been hypothesized to contribute to symptoms such as headaches, chronic fatigue, memory disturbances, and sleep disruption. However, the evidence supporting these associations remains preliminary, and further well-designed clinical studies are required to clarify their significance.^[10]^

These proposed associations between oral galvanism and oral carcinogenesis, PIOM, neurological manifestations, and systemic symptoms remain speculative. Although several biological mechanisms have been proposed, current evidence is insufficient to establish definitive causal relationships. Not all investigations have supported a direct causal relationship between oral galvanism and oral disease. Nilner et al found no significant differences in galvanic current generation, restoration quality, or mucosal conditions between symptomatic and asymptomatic individuals with metallic restorations.^[3]^ Similarly, Johansson et al likewise failed to identify significant differences in galvanic currents or taste thresholds between patients with suspected oral galvanism and healthy controls.^[7]^ Additional studies reporting similar negative or inconclusive findings are summarized in Table 2. These conflicting observations highlight the need for standardized diagnostic criteria and further prospective clinical investigations.

**Table 2:** Selected studies investigating the clinical, biological, and pathophysiological effects of oral galvanism.

Author	Year	Study design	Sample/model	Key findings
Bánóczy et al	1979	Clinical and histopathological study	1128 leukoplakia and 326 oral lichen planus patients (32 electrogalvanic lesions identified)	Thirty-two lesions were attributed to electrogalvanism, presenting clinically as leukoplakia or oral lichen planus; malignant transformation was reported in 5 cases.
Nilner et al	1982	Epidemiological study	250 individuals with extensive metallic restorations	No significant differences in galvanic current generation, restoration quality, or mucosal conditions were found between symptomatic and asymptomatic individuals.
Axéll et al	1983	Clinical observational study	68 patients with symptoms attributed to oral galvanism	No distinct clinical entity was identified, and galvanic currents did not differ from controls. However, patients demonstrated significantly lower electrical taste thresholds.
Johansson et al	1984	Comparative clinical study	54 patients with suspected oral galvanism and 15 controls	No significant differences in galvanic currents or taste thresholds were observed between patients and controls; several symptoms were attributable to other oral or stomatognathic conditions.
Hugoson	1986	Clinical observational study	100 patients (74 women and 26 men) referred for oral galvanism evaluation	Patients reported oral symptoms (metallic taste, burning sensation, xerostomia) and systemic complaints (fatigue, insomnia, concentration difficulties); however, multiple oral, dental, and medical conditions were identified as possible alternative explanations.
Sutow et al	2004	Clinical in vivo study	106 human subjects; 194 galvanic couples	Peak galvanic currents ranged from 0.24 to 102.54 μA, with newer restorations showing higher current values than older restorations. Fresh amalgams (≤6 mo) exhibited the highest currents, reaching 102.54 μA.
Procházková et al	2006	Clinical study	357 patients with metallic restorations	Patients with metallic restorations showed higher salivary concentrations of Hg, Sn, Ag, Cu, and Au. Removal of electroactive restorations reduced galvanic currents and salivary metal levels.
Wartenberg et al	2008	In vitro experimental study	UM-SCC-14-C oral cancer cells	Direct-current electrical fields (2–16 V/m) induced ROS generation, reduced cell proliferation, and increased apoptosis in oral cancer cells.
Yotova et al	2010	Case-control study	166 patients (83 with leukoplakia, 83 controls)	Patients with leukoplakia exhibited significantly higher oral electric field strengths than controls (17 vs 10 V/m; *P* < .001), suggesting an association between galvanic potentials and oral leukoplakia.
Zituni et al	2013	In vitro experimental study	*S aureus* and *E coli* cultures	Electric fields comparable with those generated by metallic restorations altered bacterial growth; *S aureus* showed greater sensitivity than *E coli*.
Korraah et al	2015	In vitro experimental study	Human leukoplakia cell line (MSK-LEUK1)	Electric fields of 1 to 10 V/m increased leukoplakia cell proliferation (10.6%–44.1%) and apoptosis (20.0%–46.6%); exposure to 16 V/m induced cellular changes resembling features of oral squamous cell carcinoma.
Chepelova et al	2023	In vitro experimental study	HaCaT-based oral mucosa-like model	Galvanic current produced greater cytotoxic and pro-inflammatory effects than corrosion ions alone.
Seo et al	2024	Retrospective study	21 PIOM patients	All patients had peri-implantitis and 95.2% had SCC; galvanic currents were proposed as a potential cofactor but were not directly measured.

*E coli* = *Escherichia coli*, HaCaT = human adult low calcium high temperature, PIOM = peri-implant oral malignancy, ROS = reactive oxygen species, SCC = squamous cell carcinoma, *S aureus* = *Staphylococcus aureus*, UM-SCC = University of Michigan–squamous cell carcinoma.

Several investigators have attempted to correlate galvanic current intensity with clinical manifestations. Ayers suggested that direct currents below 10 µA are generally not clinically significant, whereas von Fraunhofer et al reported that galvanic currents of approximately 20 µA may be associated with pain reactions. Currents ranging from 75 to 100 µA have been proposed to contribute to chronic irritation of the oral mucosa.^[8]^ However, these thresholds should be interpreted with caution because they are derived from limited studies, and their clinical applicability remains uncertain.^[10]^

## 8. Diagnosis and differential diagnosis

At present, no universally established diagnostic criteria or standardized treatment protocols exist for managing the potential adverse effects of dental metal restorations (Fig. 2).^[8,22]^ Oral galvanism can manifest through a variety of intraoral symptoms, including burning sensations, metallic taste, oral discomfort, glossodynia, and localized inflammatory changes. In some cases, lichenoid reactions have also been reported, while potential associations with oral potentially malignant disorders remain controversial.^[2]^

**Figure 2.**
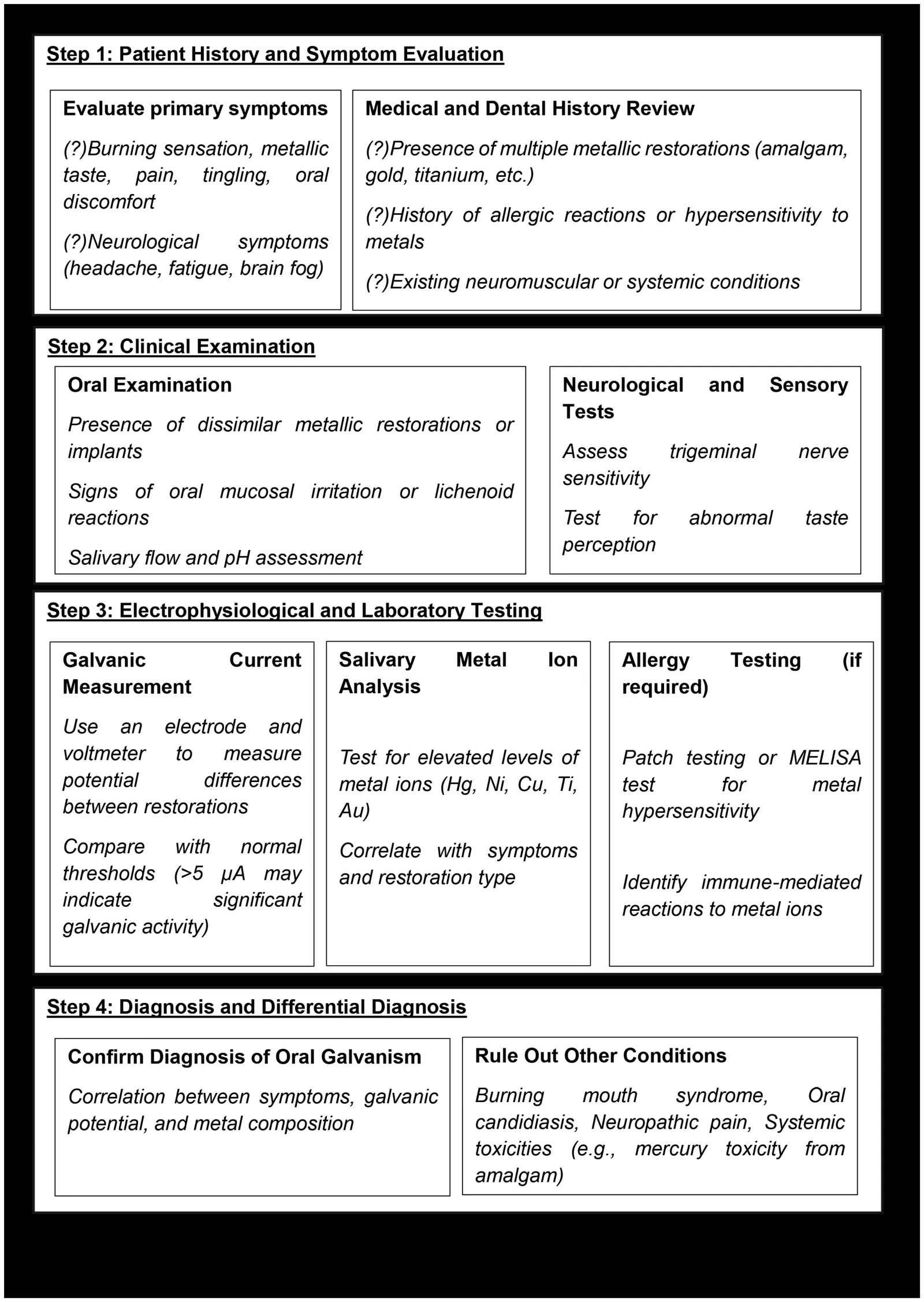
Structured clinical framework for diagnosing oral galvanism. MELISA = Memory Lymphocyte Immunostimulation Assay.

### 8.1. Clinical diagnosis

Patients typically report unexplained intraoral pain or discomfort, often arising after recent dental procedures involving metallic restorations. Given the nonspecific and variable symptoms of oral galvanism, diagnosis can be challenging. A comprehensive patient history is crucial, including details on age, gender, symptom onset and duration, general health, medical and dental history, and past or current medications^.[1,27,29]^ An intraoral examination is necessary to identify the presence, number, and surface characteristics of metallic restorations. Common clinical findings include mucosal inflammation near metal restorations and metal-induced pigmentation. These findings are categorized based on severity as none, rare, slight, moderate, or severe.^[1,31]^ However, it is essential to differentiate such pigmentation from other causes, including melanoma.^[8,15]^ A provisional diagnosis of oral galvanism can be established by isolating the metallic restorations using rubber dam application or nonmetallic barriers such as silver nitrate or composite resin. If symptom relief occurs upon separation of the dissimilar metals, this supports the diagnosis. Definitive treatment may involve replacing the existing restorations with materials of uniform composition or nonmetallic alternatives.^[1,16,20]^

### 8.2. Laboratory diagnosis

To assess systemic metal exposure, laboratory tests can measure the presence of heavy metals in urine, feces, blood, or hair samples. Commonly tested metals include lead (Pb), mercury (Hg), arsenic (As), and cadmium (Cd), while additional screening may include copper (Cu), zinc (Zn), aluminum (Al), nickel (Ni), and thallium. Hair mineral analysis has been utilized for toxicological screening, particularly for mercury exposure.^[1,2,6]^ Salivary analysis has also been explored in oral galvanism diagnosis. Alterations in salivary composition, such as changes in sodium (Na), chloride (Cl), phosphorus (P), calcium (Ca), magnesium (Mg), and immunoglobulin A (IgA) concentrations, have been reported in oral galvanism patients.^[1]^ Furthermore, decreased levels of IgA1, IgA2, and lysozyme, along with increased levels of anti-heat shock protein 60 IgA (anti-hsp60 IgA), have been observed in affected individuals. Following the removal of metallic prostheses, IgA and lysozyme levels tend to normalize, with a corresponding reduction in clinical symptoms.^[5,13]^ The Memory Lymphocyte Immunostimulation Assay test has been proposed as a diagnostic tool to assess metal hypersensitivity, helping to determine which metals a patient can tolerate and which may trigger an undesirable immune response.^[11]^

### 8.3. Electrophysiological testing

Direct measurement of galvanic potentials and currents can aid in oral galvanism diagnosis. Some investigators have proposed that galvanic currents exceeding 5 μA and voltage values ranging from 80 to 200 mV may be associated with pathological changes; however, these thresholds are derived from limited studies, and their clinical applicability remains uncertain.^[5]^ Sensibility threshold testing using an electronic pulp tester can help quantify patient response to electrical stimuli. This test involves placing the negative electrode on the occlusal surface of metallic restorations and the indifferent electrode on an adjacent tooth, allowing for voltage and current measurements.^[1]^ In addition, research suggests that galvanic currents and voltage fluctuations may impair T-lymphocyte function, affecting immune responses. Changes in potassium (K+) channel activity have been linked to the proliferation of T cells, suggesting a possible immunological component to oral galvanism pathophysiology.^[1,18]^

### 8.4. Psychiatric evaluation

Given the subjective nature of symptoms associated with oral galvanism, a psychiatric assessment may be warranted to rule out underlying psychological factors. Patients should be evaluated for symptoms such as fatigue, anxiety, depression, sleep disturbances, and reduced quality of life.^[12]^ Standardized psychological tests, including the Beck Depression Inventory, Minnesota Multiphasic Personality Inventory, State-Trait Anxiety Inventory, and Wechsler Adult Intelligence Scale, can be used for further assessment. Semi-structured psychiatric interviews may incorporate diagnostic criteria from the Diagnostic and Statistical Manual of Mental Disorders or the Cornell Medical Index health questionnaire.^[1]^

### 8.5. Differential diagnosis

Oral galvanism shares symptoms with several other conditions, necessitating careful differentiation from the following:

*BMS*: unlike oral galvanism, BMS may occur in the absence of metallic restorations and is commonly associated with systemic factors such as nutritional deficiencies, hormonal alterations, xerostomia, infections, and systemic diseases, including diabetes mellitus.^[8]^

*Oral lichenoid reactions*: oral lichenoid lesions may develop secondary to metal hypersensitivity, medications, or autoimmune disorders and can clinically resemble mucosal changes reported in oral galvanism.

*Metal hypersensitivity and allergic reactions*: certain metals, particularly nickel and mercury, may elicit hypersensitivity reactions that present with mucosal irritation, burning sensations, erythema, or ulceration, thereby mimicking manifestations attributed to oral galvanism.

*Oral pigmentation disorders*: metal-induced pigmentation should be distinguished from other causes of oral pigmentation, including melanotic macules, amalgam tattoos, and melanoma.^[8]^

*Neuropathic pain disorders*: conditions such as trigeminal neuralgia, atypical facial pain, and other neuropathic disorders may present with symptoms similar to those reported in oral galvanism and should be considered during evaluation.

## 9. Management

Managing oral galvanism involves minimizing electrochemical interactions within dental restorations while alleviating patient symptoms through appropriate clinical interventions. Treatment strategies range from modifying existing restorations to considering psychological and systemic factors.

### 9.1. Removal or replacement of electroactive restorations

Some authors have proposed that restorations associated with galvanic currents exceeding 5 μA may be electroactive and potentially associated with clinical symptoms. Similarly, currents exceeding 10 μA have been suggested as a threshold for considering restorative intervention in symptomatic patients, although the supporting evidence remains limited. Research hypothesizes that removing electroactive restorations may lead to symptom improvement in most patients within 6 months, reducing mucosal inflammation and possibly resolving lichenoid reactions. Despite the removal of the restoration, residual metallic pigmentation in oral tissues may remain over time.^[5,21]^ The highest direct-current values – up to 102 μA – have been recorded in galvanic couples involving dental amalgam and other metal alloys. Similarly, galvanic currents in titanium implant suprastructures have been reported to range from 0.5 to 12 μA/cm^2^, depending on the alloy composition in artificial saliva.^[2,9,15,19]^ These variations underscore the importance of material selection in restorative and implant dentistry.

Replacement of metal restorations may be considered in cases involving flare-ups of autoimmune diseases following restoration placement, severe burning sensations associated with metallic prostheses, positive allergy testing (patch testing or blood testing) indicating hypersensitivity to dental alloys, or the development of oral lesions that have been reported in association with oral galvanism, including lichenoid reactions and verrucous leukoplakia, particularly when alternative etiologies have been excluded.^[8,23,27]^

### 9.2. Alternative strategies for reducing galvanic effects

When direct removal of electroactive restorations is impractical, alternative techniques may help reduce galvanic interactions. Applying an insulating resin-based coating over metal restorations can help disrupt electrical conduction, thereby reducing galvanic current flow. In patients with multiple metallic restorations, replacing one of the dissimilar metals – such as substituting an amalgam filling with a ceramic alternative – can help decrease electrochemical activity.^[14,17]^

### 9.3. Psychological and systemic considerations

Some patients exhibit increased sensitivity to galvanic currents due to individual physiological and psychological factors, including immune responses, oral infections, or underlying hypersensitivities.^[10,24]^ In these cases, a multidisciplinary approach may be beneficial, integrating psychological assessments such as the Beck Depression Inventory, Minnesota Multiphasic Personality Inventory, or State-Trait Anxiety Inventory to evaluate underlying psychological contributors. Monitoring immune system responses, including alterations in salivary composition and inflammatory markers, may provide insight into biological responses potentially associated with galvanic activity, although their clinical significance remains incompletely understood.^[1,32]^

Long-term management should include periodic evaluations of occlusal stability, environmental influences, and hormonal fluctuations that may contribute to symptom persistence in patients with implant-supported restorations. Due to the multifactorial nature of oral galvanism, collaboration among dental professionals, immunologists, and mental health specialists may improve patient outcomes. Comprehensive management may involve restoration modification, psychological evaluation, and assessment of biological or immunological factors to address the multifactorial nature of oral galvanism.^[2,9,21]^

## 10. Prevention

The prevention of oral galvanism involves selecting appropriate dental materials, optimizing treatment planning, and considering individual patient factors to reduce electrochemical activity in the oral environment.

The corrosion resistance of dental alloys depends on composition, manufacturing methods, and surface treatments. Several industrial techniques have been developed to enhance corrosion resistance and minimize the risk of galvanic effects.^[8]^ Chemical passivation forms a protective oxide layer on the metal surface, significantly reducing corrosion. Citric acid passivation has been found effective in improving corrosion resistance in cobalt-chromium and titanium dental alloys.^[8,21]^ Plasma electrolytic oxidation creates a thick and stable oxide layer, enhancing metal durability and resistance to electrochemical reactions.^[18]^ Surface coatings for dental implants include methods like sol-gel surface coating, plasma spraying, chemical vapor deposition, and physical vapor deposition. These coatings not only improve corrosion resistance but also promote better osteointegration. Plasma immersion ion implantation modifies implant surfaces at a microscopic level to enhance biocompatibility and corrosion resistance.^[22,27]^ A promising surface modification method for titanium implants, providing antibacterial properties, improved corrosion resistance, and faster bone healing.^[12]^

Although these industrial approaches show short-term benefits, more long-term clinical studies are required to assess their sustained effects on bio-tribocorrosion in the oral environment.^[8]^

Dental practitioners can adopt the following preventive measures to minimize the risk of galvanic currents in restorations and implants:

Use biocompatible materials and limit the number of different metals in the oral cavity. Select metal combinations with similar galvanic potentials to minimize electrical activity. Material combinations reported to exhibit favorable galvanic compatibility include Titanium-Titanium (Ti/Ti), Titanium-Gold (Ti/Au), and Titanium-Cobalt Chromium (Ti/CoCr). Where clinically appropriate, prefer ceramic restorations on dental implants instead of metal-based prostheses to avoid galvanic interactions. Use commercially pure titanium (cp Ti) over Ti-6Al-4V alloys for implants due to its superior resistance to galvanic corrosion (Table 1).^[9,14,28]^ Prevent direct contact between dissimilar metals by using insulating barriers such as coatings, nonmetallic spacers, or cathodic inhibitors. Maintain a favorable anode-cathode surface ratio to avoid excessive electrical potential differences. Avoid acidic fluoride-containing solutions for patients with titanium implants and cobalt-chromium superstructures, as these can accelerate corrosion. Instead, daily mouthwashes with 227 ppm fluoride are preferable.^[9,16,23]^

Several lifestyle and oral hygiene factors can accelerate the corrosion of dental materials, increasing the likelihood of galvanic reactions. Patients should be advised to avoid smoking and excessive alcohol consumption, as these increase oxidative stress and metal degradation. Limit the use of ethanol-based mouth rinses and acidic oral hygiene products, which can contribute to metal ion release. Minimize the intake of acidic foods and beverages (e.g., citrus fruits, carbonated drinks, vinegar-based foods) to prevent excessive corrosion of dental restorations. Choose nonabrasive toothpastes to prevent mechanical wear on metal surfaces.^[8,17,18]^ Prevention of oral galvanism requires a multidisciplinary approach, integrating industrial advancements, careful material selection in dental practice, and patient education. By following these preventive strategies, the risk of galvanic currents can be significantly reduced, leading to improved long-term outcomes for patients with metal-based restorations and implants.

The current evidence on oral galvanism is heterogeneous, encompassing clinical observations, epidemiological investigations, and experimental studies. Given the conflicting nature of the available evidence, selected studies investigating the clinical and biological effects of oral galvanism are summarized in Table 2.^[2–5,7,22,26,30,34–38]^

Despite these preventive and management strategies, important uncertainties remain regarding the biological significance and long-term clinical implications of oral galvanism. The relevance of these uncertainties is further underscored by reports suggesting that, in certain cases, more than 30 different metallic components may coexist within a single oral cavity, including visible restorations as well as metallic structures embedded within tooth roots or concealed beneath prosthetic restorations.^[10]^ Such complex combinations of dental materials may create opportunities for electrochemical interactions whose clinical consequences are not yet fully understood. While electrochemical interactions between dissimilar metallic restorations are well established, several proposed associations with broader biological manifestations remain incompletely understood. Much of the available literature is based on experimental studies and observational reports; therefore, well-designed clinical investigations are required to clarify diagnostic thresholds, clinical relevance, and evidence-based management approaches.

## Author contributions

**Conceptualization:** Tushar Khanna.

**Investigation:** Tushar Khanna, Ashish Jain.

**Data curation:** Nakul Sharma.

**Formal analysis:** Nakul Sharma, Priyanka Saluja.

**Project administration:** Ashish Jain.

**Resources:** Gurparkash Singh Chahal, Priyanka Saluja.

**Supervision:** Gurparkash Singh Chahal, Vishakha Grover, Adel Saeed N. Alobaid.

**Software:** Anshad M. Abdulla.

**Funding acquisition:** Suraj Arora.

**Visualization:** Suraj Arora, Waled Abdulmalek Alanesi.

**Methodology:** Gotam Das, Waled Abdulmalek Alanesi.

**Writing – original draft:** Vishakha Grover.

**Writing – review & editing:** Adel Saeed N. Alobaid, Anshad M. Abdulla, Gotam Das.
